# Osteopontin adsorption to Gram-positive cells reduces adhesion forces and attachment to surfaces under flow

**DOI:** 10.1080/20002297.2017.1379826

**Published:** 2017-10-11

**Authors:** M. F. Kristensen, G. Zeng, T. R. Neu, R. L. Meyer, V. Baelum, S. Schlafer

**Affiliations:** ^a^ Department of Dentistry and Oral Health, Aarhus University, Aarhus, Denmark; ^b^ Interdisciplinary Nanoscience Center (iNANO), Aarhus University, Aarhus, Denmark; ^c^ Department of River Ecology, Helmholtz Centre for Environmental Research – UFZ, Magdeburg, Germany; ^d^ Section of Microbiology, Department of Bioscience;Aarhus University, Aarhus, Denmark

**Keywords:** Adhesion, atomic force microscopy, biofilms, caseinoglycomacropeptide, dental caries, fluorescence lectin-barcoding, fluorescence lectin-binding analysis, milk protein, osteopontin, single-cell force spectroscopy

## Abstract

The bovine milk protein osteopontin (OPN) may be an efficient means to prevent bacterial adhesion to dental tissues and control biofilm formation. This study sought to determine to what extent OPN impacts adhesion forces and surface attachment of different bacterial strains involved in dental caries or medical device–related infections. It further investigated if OPN’s effect on adhesion is caused by blocking the accessibility of glycoconjugates on bacterial surfaces. Bacterial adhesion was determined in a shear-controlled flow cell system in the presence of different concentrations of OPN, and interaction forces of single bacteria were quantified using single-cell force spectroscopy before and after OPN exposure. Moreover, the study investigated OPN’s effect on the accessibility of cell surface glycoconjugates through fluorescence lectin-binding analysis. OPN strongly affected bacterial adhesion in a dose-dependent manner for all investigated species (*Actinomyces naeslundii*, *Actinomyces viscosus*, *Lactobacillus paracasei* subsp. *paracasei*, *Staphylococcus epidermidis*, *Streptococcus mitis*, and *Streptococcus oralis*). Likewise, adhesion forces decreased after OPN treatment. No effect of OPN on the lectin-accessibility to glycoconjugates was found. OPN reduces the adhesion and adhesion force/energy of a variety of bacteria and has a potential therapeutic use for biofilm control. OPN acts upon bacterial adhesion without blocking cell surface glycoconjugates.

## Introduction

Targeting bacterial adhesion to solid surfaces is a promising approach to prevent biofilm-related disease [1–3]. This particularly holds true for the oral cavity where the long-standing presence of biofilms on teeth is the main cause of dental caries and periodontitis [4,5]. At the same time, the commensal oral microbiota, residing on mucosal surfaces, protects the host against opportunistic pathogens and makes a relevant contribution to human physiology. The widespread application of antimicrobial agents in toothpastes and mouthwashes [6] may affect bacterial viability in disease-related biofilms to some extent, but it has an even greater impact on the commensal microbiota. Therapeutic approaches that target bacterial adhesion to dental tissues rather than bacterial viability could reduce or delay biofilm formation without affecting microbial homeostasis to the same extent.

It was found that the phosphoprotein osteopontin (OPN) from bovine milk interferes with the adhesion of the early dental colonizer *Streptococcus mitis* to saliva-coated surfaces [7], and that it reduces biofilm formation in a multi-species model biofilm dominated by *S. mitis* [8]. Milk OPN is an intrinsically disordered protein, with little secondary structure and a high degree of phosphorylation [9]. It is present in elevated concentrations in human breast milk (~140 mg/L) [10] and has been found to have a positive influence on the development of the human immune system [11], as well as on the intestinal gene expression in rhesus monkeys [12]. OPN was shown to bind different strains of *Staphylococcus aureus* and *Streptococcus agalactiae* and opsonize them for phagocytosis [13], but except for this finding and the results mentioned above, little is known about the interaction between osteopontin and bacteria. In particular, it is not yet understood how OPN interferes with adhesion, and if the effect is a general phenomenon that applies to other bacteria in the oral cavity. The present study employed a representative selection of organisms that are among the earliest colonizers of the tooth surface and organisms that are involved in the caries process, including strains from the genera *Actinomyces*, *Bifidobacterium, Lactobacillus*, *Streptococcus*, and *Rothia* [14]. The study quantified how different concentrations of OPN affected the colonization of saliva-coated surfaces by these organisms and determined the effect of OPN on the adhesion force of selected bacteria using single-cell force spectroscopy (SCFS).

The mechanisms by which bacteria adhere to saliva-coated surfaces are complex and manifold. An array of different adhesins and corresponding receptors has been described for streptococci [15] and *Actinomyces* spp. [16], whereas data on the adhesion of lactobacilli to salivary receptors are scarce. Many adhesins belong to the superfamily of cell wall–anchored polypeptides with the C-terminal consensus motif LPxTz, including type I fimbriae of *Actinomyces* spp. [17], the streptococcal antigen I/II [18], and serine-rich repeat protein (SRRP) families [19–23]. In SRRP adhesins, which have been identified in both streptococci and lactobacilli [23], glycoconjugates play an important role for adhesion [21,24]. Moreover, glycoconjugates are essential structural elements of lipoteichoic acids [25], which also mediate adhesion to saliva [26,27]. It was hypothesized that OPN interferes with adhesion by interacting with glycoconjugates on the cell surface, and therefore the ability of OPN to prevent the binding of fluorescently labeled lectins competitively to cells of *S. mitis*, *Actinomyces naeslundii*, and *Lactobacillus paracasei* subsp. *paracasei* was assessed.

Targeting bacterial adhesion could be a valuable approach to disease control in several medical fields, and therefore strains of *Staphylococcus epidermidis* and *Enterococcus faecalis*, involved in medical device-related infections [28], were included in the adhesion experiments. As the polysaccharide intercellular adhesin (PIA) is an important factor in biofilm formation of *S. epidermidis* [29], the organism was included in the lectin-binding analysis.

## Materials and methods

### Bacterial strains

*Actinomyces naeslundii* AK 6, *Actinomyces viscosus* CCUG 33710, *E. faecalis* DSM 20478, *L. paracasei* subsp. *paracasei* DSM 20020, *S. epidermidis* 1457, *S. epidermidis* 1585, *S. mitis* SK 24, *Streptococcus mutans* DSM 20523, and *Streptococcus oralis* SK 248 were grown aerobically at 35°C on Columbia blood agar (Statens Serum Institut, Copenhagen, Denmark). *Bifidobacterium dentium* DSM 20436 and *Rothia dentocariosa* DSM 43762 were cultivated anaerobically at 35°C on chocolate agar or Columbia blood agar, respectively. Prior to experimental use, all organisms were grown at 35°C in 5 mL of Todd–Hewitt broth (THB; Roth, Karlsruhe, Germany) until early stationary phase.

### Binding of OPN to bacterial cells

To investigate the binding of OPN to bacterial cell surfaces, the protein was fluorescently labeled. OPN was dissolved in 3 mL of NaHCO_3_ buffer (50 mM; pH 9.5) at room temperature, and fluorescein isothiocyanate (FITC; Sigma–Aldrich, Brøndby, Denmark) in dimethyl sulfoxide was added dropwise within 30 min, yielding a final molar OPN/FITC ratio of 1/10. After magnetic stirring for 5 h, the labeled protein was purified by dialysis for 48 h (3 mL against 1,000 mL) in a dialysis tube with a molecular weight cutoff of 3.5 kDa (Spectra/Por® RC; Spectrum Labs, Rancho Dominguez, CA). Then, labeled OPN was freeze-dried for 48 h (Triad cascade benchtop freeze dry system; Labconco Corp., Kansas City, MO). Bacteria were washed in phosphate-buffered saline (PBS; 4,696 g), adjusted to an OD_550_ of 0.1, incubated with labeled OPN (20 µM/L) at 35°C for 15 min, washed twice with PBS to remove unbound protein, and counterstained with SYTO® 60 (10 µM; Thermo Fisher Scientific, Naerum, Denmark). A confocal microscope (Zeiss LSM 700; Carl Zeiss, Jena, Germany) equipped with a 63× oil immersion objective (alpha Plan-Apochromat; Carl Zeiss) was used for image acquisition. FITC and SYTO® 60 were excited at 488 and 639 nm and detected with 640 nm short- and long-pass filters, respectively.

### Adhesion experiments

Sterile human saliva was prepared according to the method of de Jong et al. [30] and titrated to pH 7. OPN (Lacprodan® OPN-10; Arla Foods, Ingredients Group P/S, Viby, Denmark; MW 23.3 KDa, 99.5% purity) and caseinoglycomacropeptide (Lacprodan® CGMP-20, Arla Foods Ingredients Group P/S, Viby, Denmark; 7.5 KDa, 98% purity) were titrated to pH 7 in PBS and pasteurized for 20 min at 80°C. Bacterial suspensions were centrifuged (4,696 *g* for 5 min), washed once in THB, and resuspended in THB to an OD_550_ of 0.5. Oral bacteria were then diluted in saliva and PBS (one part bacteria, three parts saliva, six parts PBS with OPN or CGMP), resulting in final concentrations of 0, 0.46, 4.6, 46, or 460 µM of OPN, or 460 µM of CGMP. *E. faecalis* and *S. epidermidis* were prepared in the same way but without the addition of saliva (one part bacteria, nine parts PBS).

Bacterial adhesion was tested in a microfluidic device with a polydimethylsiloxane (PDMS) surface, providing shear-controlled flow (Bioflux EZ; fluxion Biosciences, San Franscisco, CA). For adhesion of the oral organisms, channels were flushed with saliva diluted in PBS (one part saliva, two parts PBS) using reverse flow (2 min; 1 dyn/cm^2^) followed by 30 min of static incubation to facilitate saliva-coating of the PDMS surface. For *E. faecalis* and *S. epidermidis*, channels were conditioned with PBS. Subsequently, bacterial suspensions were pumped through the channels at a flow rate of 9.45 µL/h, corresponding to 0.1 dyn/cm^2^, for 1 h at 35°C. Thereafter, non-adherent cells were removed by 20 min of PBS flow (10 min, 1 dyn/cm^2^; 10 min, 0.1 dyn/cm^2^). The adhesion experiments were carried out in two series: one testing the effect of OPN at different concentrations (0, 0.46, 4.6, and 46 µM), and one comparing the effect of OPN and CGMP at very high concentrations (460 µM).

It was hypothesized that OPN blocked glycoconjugate-mediated adhesion, and therefore the effect of OPN on adhesion of *S. epidermidis* 1585 was tested, which does not contain the operon for production of the polysaccharide intercellular adhesin (PIA). Adhesion in the absence and presence of 460 µM of OPN or CGMP was tested for this strain.

### Quantification of bacterial adhesion

For each channel, nine microscopic fields of view (FOV; 1,920 × 1,440 pixels in size) were imaged in random locations with a bright-field microscope (Zeiss Axio Vert A1; Carl Zeizz) equipped with a 40×/0.75 NA objective (EC Plan-NEOFLUAR; Carl Zeiss) and a CCD camera (Zeiss AxioCam ERc5s; Carol Zeiss). The experiments were performed in technical duplicates (two flow channels) and biological duplicates. All images were cropped to a size of 960 × 720 pixels in ImageJ [31] to remove background noise at the image borders. Then images were imported into the digital image analysis software daime [32] and segmented with an appropriate brightness threshold, and the area covered by bacterial cells was quantified.

### AFM SCFS

*A. naeslundii*, *L. paracasei* subsp. *paracasei*, *S. mitis*, and *S. epidermidis* 1457 were included in the experiments. Bacteria were grown until late exponential phase at 35°C in THB or, for *S. epidermidis*, in Tryptic Soy Broth (Scharlau, Barcelona, Spain), harvested by centrifugation (4,696 *g* for 5 min), washed, and re-suspended in PBS. A drop of the bacterial suspension was placed on a glass slide (SuperFrost Ultra Plus; Thermo Fisher Scientific) and allowed to settle for 5–10 min without drying out. The bacteria attached better to the glass surface when harvested in late exponential instead of early plateau phase. Optimal bacterial density on the slide was reached when there were >10 single cells in a 100 µm square where the AFM scanner was free to move around. An AFM probe CSC38/TIPLESS/Au (MikroMasch, Sofia, Bulgaria) was coated with either saliva (for oral bacteria) or hydrophobic thiol (for *S. epidermidis*) before it was mounted on Nanowizard 4 AFM (JPK Instruments, Berlin, Germany). For saliva coating, AFM probes were incubated for 15 min at room temperature in sterile saliva (1:3 in PBS; pH 7) and then washed with PBS. For hydrophobic coating, AFM probes were incubated in 1-Dodecanethiol (1 mM; Sigma–Aldrich) overnight and washed with ethanol and MilliQ water. The cantilever was then positioned with the very end above the cell, ensuring contact with the cell. A detailed protocol for single-cell contact with tipless cantilevers has been published previously [33]. Briefly, the cantilever was approached to a single cell until a specified force set point was reached and then retracted. The cantilever deflection versus Z piezo displacement was recorded and converted to a force–distance curve (force curve) after calibration of sensitivity and spring constant, following the standard procedure of data processing (see below). The following settings were used throughout the measurements: 1 nN set point, 1 µm Z length, 2 s contact time, 1 µm/s speed, 20 force curves per cell, and more than five cells per sample. After the measurement, the AFM head was removed from the sample, and the sample was incubated in OPN solution (460 µM) for 15 min and washed, and the same measurement was repeated. The DirectOverlay^TM^ (JPK Instruments) function was used to reproduce the positions of the AFM measurements, so that the same cells were measured before and after treatment. All measurements were performed at least twice with independently grown cultures and fresh AFM probes.

Force curves were analyzed by JPK Data Processing software (JPK Instruments). Calibration of photodiode sensitivity was done by performing force spectroscopy on glass slides right before measurements on cells, and the spring constant was calibrated by thermal tuning. Deflection versus Z curves were then converted to force–distance curves. Adhesion force was defined by the largest force during retraction, and adhesion energy was calculated by the integration of the retraction part of the force curves (i.e. the work needed to pull the cell off the cantilever).

### Fluorescence lectin bar coding

Based on the results of the adhesion experiments, *A. naeslundii*, *L. paracasei* subsp. *paracasei*, *S. epidermidis* 1457, and *S. mitis* were chosen for fluorescence lectin bar coding [34] and binding analysis. For this purpose, the binding of 76 fluorescently labeled lectins (FITC or Alexa Fluor® 488) to these bacteria was tested (see Supplementary Table S1 for details). The cells were washed twice with sterile PBS (4,696 *g* for 5 min), adjusted to an OD_550_ of 0.1, and then immobilized on nitrocellulose filters (0.45 µm; 1 mL of bacterial suspension/filter) using a vacuum filtering kit with hand pump. Filters were placed on microscopic slides and stained with one lectin each (0.9 µL; 100 µg/mL). The slides were stored at room temperature in the dark for a minimum of 20 min and rinsed three times with tap water using a pipette and absorbing triangles (Fine Science Tools, Heidelberg, Germany) prior to microscopy analysis. Images were acquired with an upright confocal microscope (TCS SP5X; Leica, Wetzlar, Germany) equipped with 63×/1.2 NA water immersion objective, controlled by the software LAS AF v2.7.3.9 (Leica). A supercontinuum white laser was used for excitation (490 nm), and emission was detected from 550 to 570 nm. Based on visual examination, lectins showing a strong binding were chosen for subsequent fluorescence lectin-binding analysis (FLBA) experiments.

### FLBA

The following lectins were included in the FLBA. *A. naeslundii: Bananas musa acuminate* (BanLec-FITC), Concanavalin A (ConA-FITC), *Vicia graminea* (VGA-FITC), and *Triticum vulgare* (WGA-FITC). *L. paracasei* subsp. *paracasei: Agaricus bisporus* (ABA-FITC) and *Helix pomatia* (HPA-FITC). *S. epidermidis*: WGA. *S. mitis*: VGA and WGA. Bacterial suspensions were washed twice with sterile PBS (4,696 *g* for 5 min), adjusted to an OD_550_ of 0.1, and immobilized for 15 min on microscopy slides (SuperFrost Ultra Plus; Thermo Fisher Scientific) or in plasma-treated flow cells (µ-slide VI ibitreat; ibidi, Planegg/Martinsried, Germany). After rinsing with PBS, cells were stained with the respective lectins, incubated in the dark for 30 min, and rinsed with PBS to remove unbound lectins. The effect of OPN on lectin binding was tested by adding OPN to a concentration of 460 µM during lectin binding followed by washing with PBS or PBS with 460 µM of OPN. After lectin staining, bacterial cells were counterstained with SYTO® 60 (10 µM; Thermo Fisher Scientific).

### Quantification of fluorescent lectin binding

For each lectin–bacteria combination, six randomly chosen FOV were imaged with a confocal microscope (Zeiss LSM 700; Carl Zeiss) equipped with a 100×/1.46 NA oil immersion objective (alpha Plan-Apochromat; Carl Zeiss). FITC and SYTO® 60 were excited with 488 and 639 nm lasers, and detected through 640 nm short- and long-pass filters, respectively. Excitation of FITC was performed with fixed settings for laser power and gain. Experiments were performed in biological triplicates. Images were exported into the software daime, and SYTO® 60 images were segmented to identify bacterial cells (objects). The resulting object mask was transferred to the FITC images, and the average fluorescence intensity of all objects was determined in each image.

### Statistical analyses

Bacterial adhesion data were analyzed by a three-level mixed effects linear regression, with the area covered by bacterial cells as the dependent variable, the treatment in each experimental series (PBS, 460 µM CGMP, 460 µM OPN or 1 effect, and FOV (*n* = 9), technical replicate (*n* = 2), and biological replicate (*n* = 2) as the levels. Student’s *t*-tests were employed to analyze differences between mean values of the single-cell adhesion force and adhesion energy (paired *t*-test) and average fluorescence intensity resulting from lectin binding (unpaired *t*-test). Normal distribution of adhesion forces, adhesion energies, and average fluorescence intensities were verified using qq-plots and Shapiro–Wilk tests. For all analyzes, *p*-values <0.05 were considered to indicate statistical significance.

## Results

**Figure 1.**
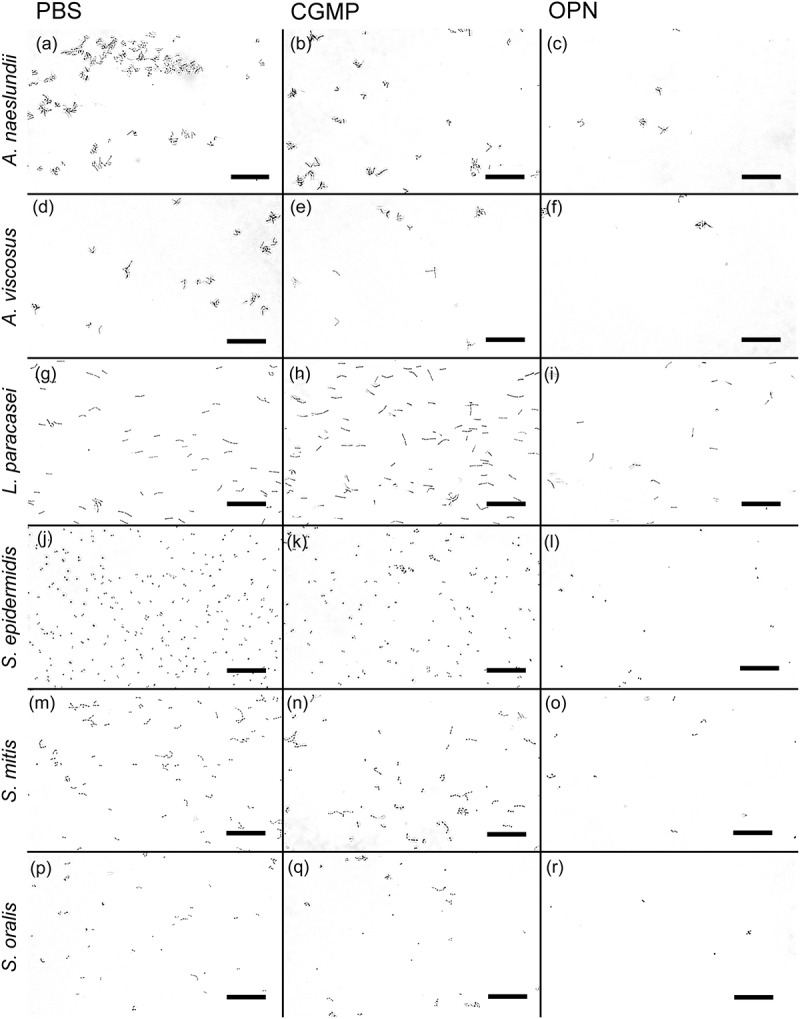
Representative bright field images of adhering bacteria. Adhesion was tested under flow (9.45 µL/h) in the presence of saliva and phosphate-buffered saline (PBS; left column), 460 µM of caseinoglycomacropeptide (CGMP; middle column), or 460 µM of osteopontin (OPN; right column). Considerably fewer cells of *Actinomyces naeslundii* (a–c), *Actinomyces viscosus* (d–f), *Lactobacillus paracasei* subsp. *paracasei* (g–i), *Staphylococcus epidermidis* (j–l), *Streptococcus mitis* (m–o), and *Streptococcus oralis* (p–r) adhered in the presence of OPN compared to CGMP and control treatment with PBS. In experiments with *S. epidermidis*, saliva was omitted. Bars = 20 µm.

**Figure 2.**
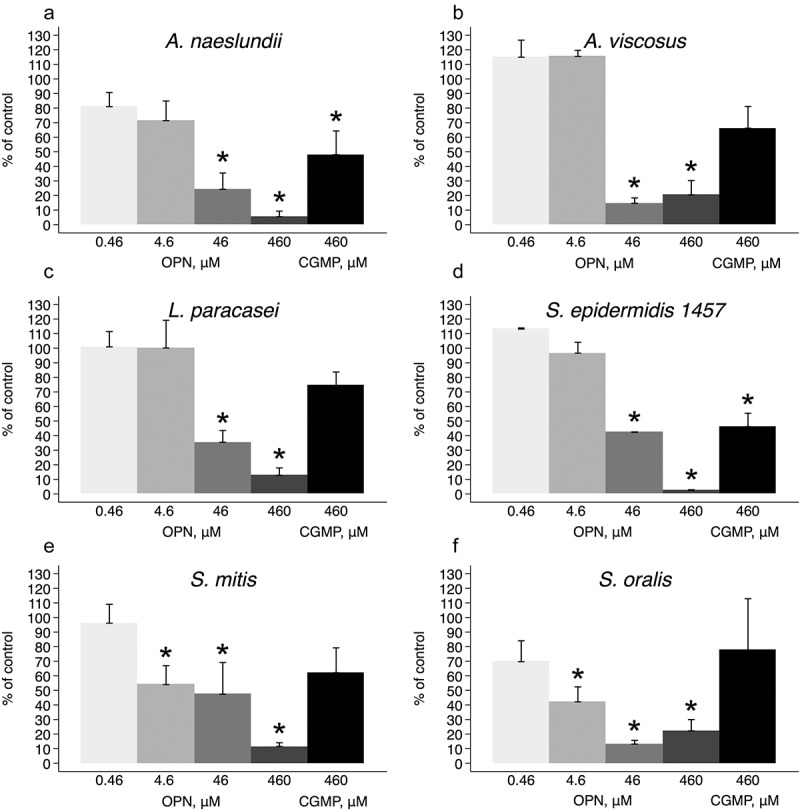
Dose-dependent effect of OPN on bacterial adhesion. Adhesion of *A*. *naeslundii* (a), *A*. *viscosus* (b), *L*. *paracasei* subsp. *paracasei* (c), *S*. *epidermidis* (d), *S*. *mitis* (e), and *S*. *oralis* (f) under flow was significantly reduced by 460 µM and 46 µM OPN compared to control treatment with PBS. At 4.6 µM OPN, only *S. mitis* and *S. oralis* were affected; at 0.46 µM OPN, the effect ceased. CGMP (460 µM) had a weak effect on adhesion that was only statistically significant for *S. epidermidis* and *A. naeslundii*. Error bars = standard errors of mean. *p < 0.05.

OPN bound to the surfaces of all investigated bacterial species, as shown by incubation with fluorescently labeled OPN (Supplementary Figure S1). OPN had a strong, dose-dependent effect on the adhesion of *A. naeslundii*, *A. viscosus*, *L. paracasei* subsp. *paracasei*, *S. epidermidis* 1457, *S. mitis*, and *S. oralis* (Figures 1 and 2). At concentrations of 460 or 46 µM of OPN, the number of bacteria adhering to the flow cell was significantly lower for all strains tested compared to control treatment with PBS (*p* < 0.05). At 4.6 µM, OPN impaired adhesion of *S. oralis* and *S. mitis* (*p* < 0.05) but not *A. naeslundii*, *L. paracasei* subsp. *paracasei*, and *S. epidermidis*. OPN exerted no significant effect on any of the strains at 0.46 µM. Like OPN, CGMP is a milk phosphoprotein that was previously reported to hamper bacterial adhesion to saliva-coated surfaces [35,36], and it was therefore used in this study to determine if the effect of OPN might extend to phosphoproteins in general. At a concentration of 460 µM, CGMP had a moderate effect on bacterial adhesion that was statistically significant for *A. naeslundii* and *S. epidermidis*. However, OPN’s effect on adhesion of these strains was more pronounced (*p* < 0.05). *B. dentium*, *R. dentocariosa*, *S. mutans*, and *E. faecalis* were included in the experiments, as they play a role in the caries process and in medical device–related infections, but none of these bacteria attached to the saliva-coated surface, irrespective of the presence of OPN (data not shown). These strains were therefore omitted from subsequent analyses.

**Figure 3.**
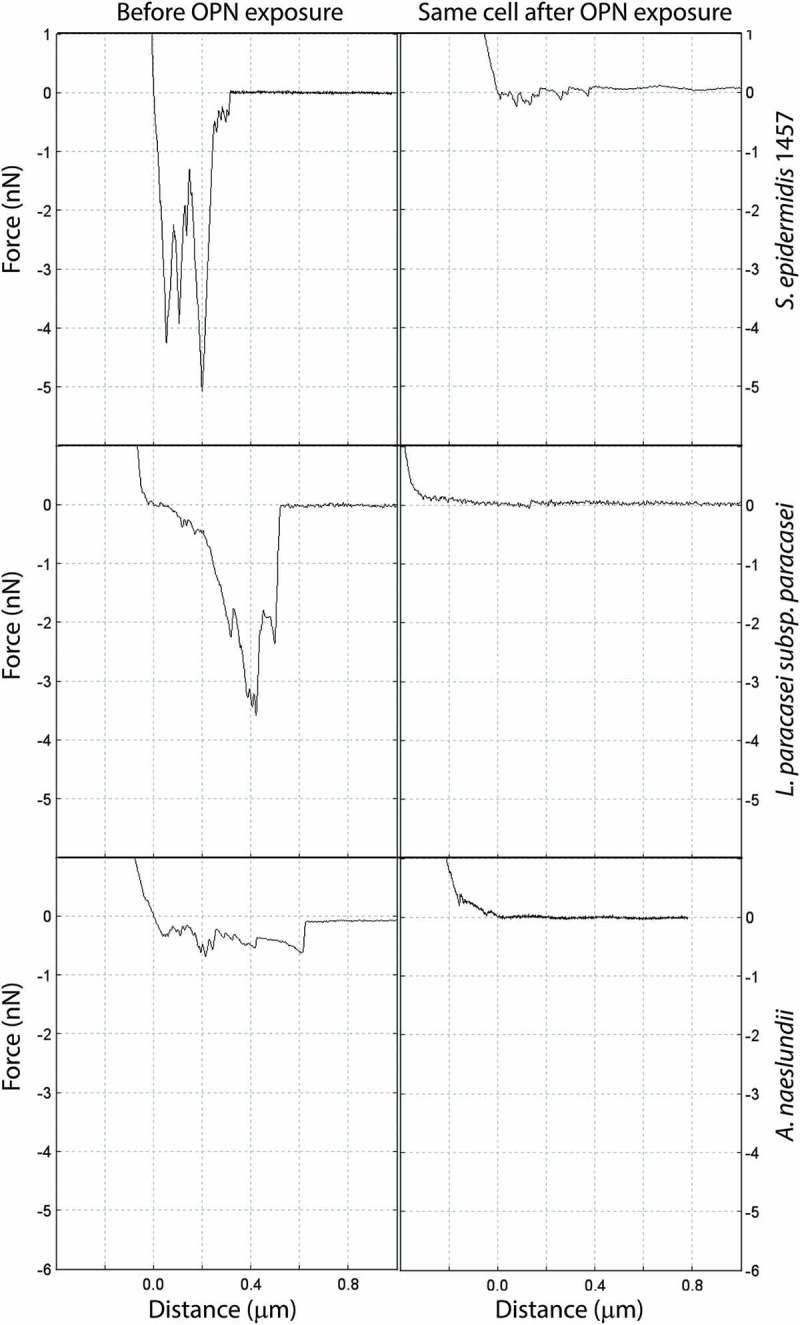
Force–distance curves for single cells before and after OPN exposure. Adhesion of single bacterial cells to coated AFM cantilevers was measured by force spectroscopy before and after exposure to 460 µM of OPN solution. Graphs show retraction parts of representative force–distance curves.

Attachment of bacteria to surfaces under flow relies on adhesion forces that exceed the lateral shear forces that would otherwise detach the cell from the surface. To understand how OPN affects the interaction forces between bacteria and substrate, adhesion force and adhesion energy of single cells were measured before and after OPN exposure (Table 1 and Figure 3). Force–distance curves obtained prior to OPN addition showed that *S. epidermidis* and *L. paracasei* subsp. *paracasei* were highly adhesive, with maximum adhesion forces of up to 5 nN, while *A. naeslundii* had lower maximum adhesion force but comparable adhesion energy due to the much longer rupture length. The rupture length indicates how far adhesive biomolecules extend from the cell surface before the bond with the AFM cantilever ruptures during retraction of the cantilever from the cell. The long rupture length of *A. naeslundii* is likely due to fimbriae extending from the cell surface. OPN treatment lowered the average maximum adhesion force to ≤0.5 nN for all three strains tested. The adhesion energy more than halved for *A. naeslundii*, and it decreased by >80% for *S. epidermidis* and *L. paracasei* subsp. *paracasei* (Table 1). The absence of adhesion peaks after OPN treatment suggests that the adhesins previously exposed on the cell surface are covered by OPN and are therefore unable to interact with their salivary ligands (*A. naeslundii* and *L. paracasei* subsp. *paracasei*) or with the hydrophobic cantilever tip (*S. epidermidis*).

**Table 1.** Effect of osteopontin (OPN) on bacterial adhesion force and energy.

	Adhesion force (nN)		Adhesion energy (fJ)	
Strain	PBS, *M* (*SD*) *n*	OPN, *M* (*SD*) *n*	PBS, *M* (*SD*) *n*	OPN, M (*SD*) *n*
Actinomyces *naeslundii*	0.92 (0.78) 179	0.41 (0.32) 175	0.145 (0.210) 179	0.056 (0.082) 175
	0.92 (0.35)^§^ 9	0.41 (0.13) 9	0.144 (0.127) 9	0.056 (0.030) 9
*Lactobacillusparacasei*	2.33 (1.45) 281	0.46 (0.71) 337	0.574 (0.710) 281	0.059 (0.117) 337
	2.40 (1.01)* 16	0.47 (0.47) 16	0.690 (0.729)^#^ 16	0.059 (0.050) 16
*Staphylococcusepidermidis*	1.77 (1.12) 376	0.31 (0.69) 364	0.193 (0.170) 376	0.025 (0.100) 364
	1.76 (0.79)* 19	0.35 (0.31) 18	0.193 (0.134)* 19	0.029 (0.039) 18

Adhesion force and energy of single bacterial cells were quantified with single-cell force spectroscopy before and after treatment with 460 µM of OPN. Lower line estimates are based on aggregated data – mean values of replicate measurements for single cells.

*p < 0.0001; ^§^ *p* < 0.001; ^#^ *p* < 0.002.

PBS, phosphate-buffered saline; *SD*, standard deviation.

**Figure 4.**
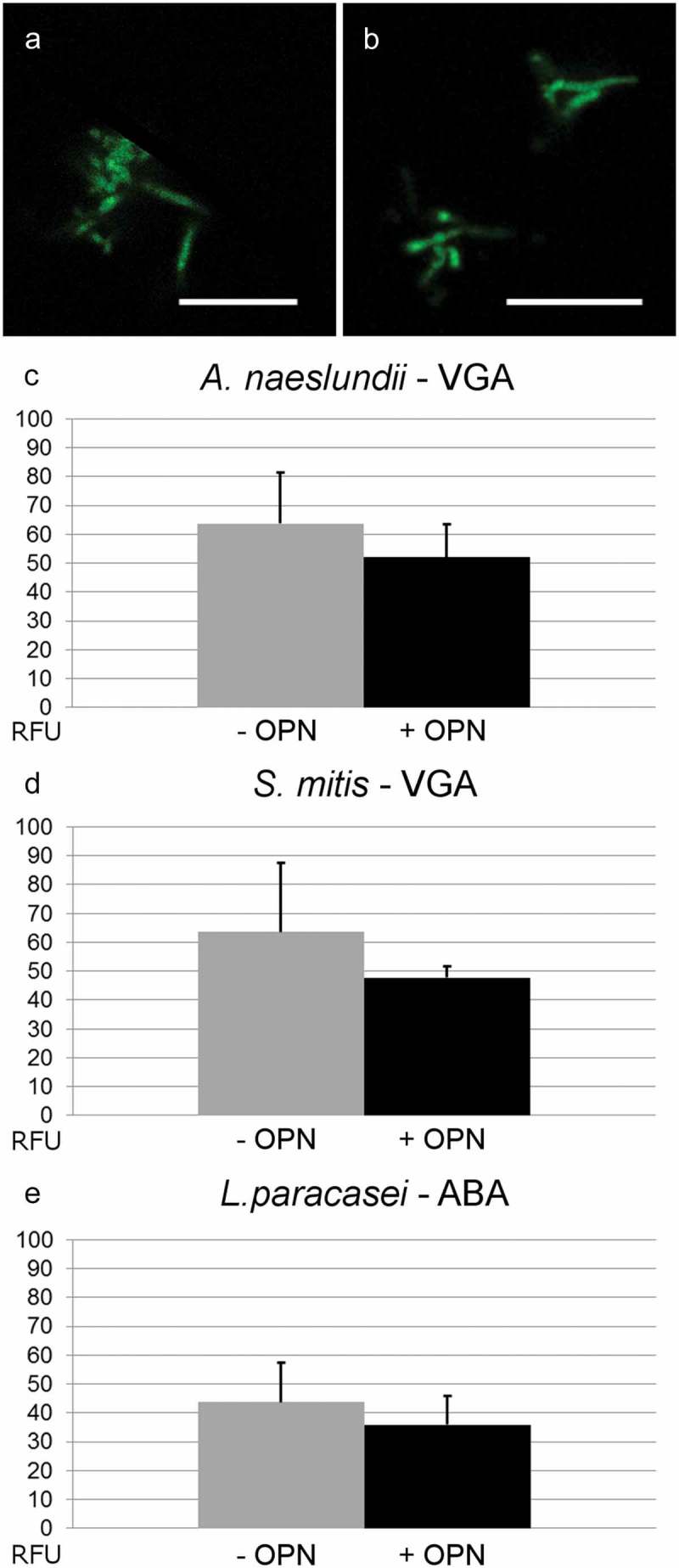
Fluorescent lectin binding to bacteria in the presence or absence of OPN. Bacteria were immobilized and incubated with selected fluorescently labeled lectins in the presence or absence of 460 µM of OPN. Images with fixed laser settings were acquired, and the average fluorescent intensity of the bacteria (RFU = relative fluorescence units) was determined. (a and b) Representative images of *A*. *naeslundii* without (a) and with (b) OPN. Bars = 10 µm. (c**–**e) For all three combinations, *A. naeslundii* and *Vicia graminea* lectin (VGA), *S*. *mitis* and VGA, and *L*. *paracasei* subsp. *paracasei* and *A*. *bisporus* lectin (ABA), the fluorescent intensities were lower in the presence of OPN, but the differences were not statistically significant.

Extracellular polysaccharides and cell wall–attached glycoconjugates contribute to adhesion and biofilm formation in many biofilm-forming bacteria, and it was hypothesized that OPN prevented adhesion by blocking the interactions of such adhesins on the cell surface. To test this hypothesis, the study investigated if OPN (460 µM) reduced the binding of fluorescently labeled lectins. The lectin screening identified four lectins suitable for binding to *A. naeslundii* (BanLec, ConA, VGA, and WGA), two lectins each for *L. paracasei* subsp. *paracasei* (ABA and HPA) and *S. mitis* (VGA, WGA), and only WGA for *S. epidermidis* 1457. OPN had no effect on the binding of BanLec, ConA, and WGA to *A. naeslundii*, of HPA to *L. paracasei* subsp. *paracasei*, and of WGA to *S. mitis* and *S. epidermidis* (data not shown). Slight reductions in VGA binding to *A. naeslundii* and *S. mitis* and in ABA binding to *L. paracasei* subsp. *paracasei* were observed as a result of OPN exposure (Figure 4). However, none of these were statistically significant, indicating that OPN is unable to block the diffusion of lectins to glycoconjugates. To address further if OPN’s effect was linked to blocking polysaccharide-mediated adhesion, the effect of OPN on *S. epidermidis* 1457 and the polysaccharide-deficient strain *S. epidermidis* 1585 was compared. OPN did reduce attachment of the polysaccharide-deficient strain (Supplementary Figure S2), suggesting that OPN prevents attachment through a different mechanism.

## Discussion

Previous studies have shown that OPN binds to the cell surface of *S. mitis* [7], a pioneer colonizer of dental enamel, which is also associated with the development of caries lesions [37–41]. The presence of OPN hampers adhesion of *S. mitis* to saliva-coated surfaces and diminishes biofilm formation in a multi-species *in vitro* oral biofilm model dominated by *S. mitis* [8]. This study now shows that OPN binds to a variety of other Gram-positive species (Supplementary Figure S1), suggesting that OPN binds to the cell surface through an unspecific mechanism or through specific binding to cell surface components that most Gram-positives have in common, such as polysaccharides or teichoic acids.

OPN adsorption dramatically reduced the adhesion forces between bacterial cells and saliva-coated or -uncoated surfaces (Table 1 and Figure 3), resulting in up to 92% fewer cells colonizing salivary-coated surfaces under flow (Figure 2). The effect of OPN on bacterial adhesion was dose and species dependent. Adhesion of streptococci (*S. oralis* and *S. mitis*) was most severely affected, and effects of OPN on these species could be measured at concentrations down to 4.6 µM. This finding corroborates results from a previous study where *A. naeslundii* was less affected by 26.5 µM of OPN than *S. mitis*, albeit in a different flow cell system [7]. The difference between the bacterial species can either reflect differences in their affinity for OPN adsorption or differences in how OPN intercepts their specific mode of attachment. *A. naeslundii* might be less susceptible to OPN treatment due to the presence of type I/II fimbriae that extend up to 700 nm from the cell surface [17] and thereby potentially penetrate the adsorbed OPN layer. *A. viscosus*, *Lactobacillus* spp., and streptococci can also produce fimbriae or pili for adhesion [42–46], but the presence and length of such cell surface appendages vary considerably, even between different strains of the same species, and there is no information about pili or fimbriae production by the strains employed in this study. Alternatively, the observed differences might result from differential blocking of salivary receptors by OPN at low concentrations. Type I fimbriae from *A. naeslundii* preferentially bind proline-rich proteins [16], whereas streptococcal antigen I/II and SRRP adhesins interact with gp340 and sialic acid residues in glycoproteins, respectively [15]. *S. epidermidis* was included in this study to broaden the scope of the analyses beyond the oral cavity. *S. epidermidis* is responsible for the majority of nosocomial infections, as it manages to survive as biofilms on surfaces in the hospital environment [28]. OPN strongly reduced the adhesion of *S. epidermidis* to PDMS, demonstrating that adsorption of OPN to bacteria not only blocks their interaction with saliva but also prevents attachment to abiotic surfaces.

It was hypothesized that OPN adsorbed to the cell surface blocks the interactions of glycoconjugate and polysaccharide adhesins, as such an effect would result in the response that was observed. However, this could not be confirmed. OPN prevented adhesion of the polysaccharide-negative *S. epidermidis* 1585 to a similar extent as the polysaccharide-producing *S. epidermidis* 1457 (Supplementary Figure S1). Furthermore, lectin-based staining of glycoconjugates on the cell surface was unaffected by OPN (Figure 4), indicating that polysaccharide and glycoconjugate adhesins remained accessible to their ligands, even if they are not functional in cell attachment. Hence, OPN did not specifically intercept the polysaccharide/glycoconjugate–ligand interaction, but more likely provided a steric barrier that prevented the accessibility of polysaccharides and other adhesins on the cell surface. Single-cell force spectroscopy also pointed to a general mechanism through steric hindrance. The three strains tested are equipped with different sets of adhesins used for attachment to abiotic surfaces (*S. epidermidis*) and saliva-coated surfaces (*A. naeslundii* and *L. paracasei*), and the abolishment of attachment peaks after OPN exposure suggests that whatever the adhesins were, OPN adsorption blocked their interaction.

In conclusion, bioactive proteins derived from dairy sources may represent a promising avenue to delay biofilm formation without eradicating the commensal bacteria of the mouth. Both lactoferrin and different preparations of casein, including CGMP, have been shown to reduce bacterial adhesion to salivary-coated surfaces [35,36,47]. The present study, however, shows that OPN’s effect on adhesion by far exceeds the one of CGMP. OPN targets a broad range of Gram-positive bacteria, with a pronounced effect on streptococcal species, which might prove advantageous in a clinical context, as streptococci are strongly associated with dental caries [48]. Further studies testing the effect of OPN on bacterial adhesion *in situ* are required to determine its potential use as an anti-biofilm agent for caries control.

## Supplementary Material

Supplemenatal_data.zip
