# Supplementary material for: Osteopontin adsorption to Gram-positive cells reduces adhesion forces and attachment to surfaces under flow
Source: J Oral Microbiol. 2017 Oct 11;9(1):1379826. doi: 10.1080/20002297.2017.1379826 (PMC5646589; doi:10.1080/20002297.2017.1379826)
Supplement: Supplemenatal_data.zip [file ZJOM_A_1379826_SM6151.zip › Supplementary-Legends.docx]

Table S1. Fluorescently labelled lectins employed during the lectin screening. The table shows the abbreviations, isolation sources, fluor conjugates, suppliers and carbohydrate specificities for all lectins employed in the study.

Figure S2. Binding of fluorescently labelled osteopontin (OPN) to bacterial cells. Cells of *Actinomyces naeslundii* (**A**), *Actinomyces viscosus* (**B**), *Lactobacillus paracasei* subsp. *paracasei* (**C**), *Staphylococcus epidermidis* (**D**), *Streptococcus mitis* (**E**) and *Streptococcus oralis* (**F**) were incubated with fluorescein isothiocyanate-labelled OPN, washed and then imaged with a confocal microscope. OPN bound to the surface of all investigated bacterial species. Bars = 10 µm.

Figure S3. Effect of osteopontin (OPN) on *Staphylococcus epidermidis* 1585. **A**) OPN binds to cells of *S. epidermidis* 1585, which does not produce polysaccharide intercellular adhesin (PIA)**.** Bar = 10 µm. **B**) Adhesion of *S. epidermidis* 1585 under flow was significantly reduced by 460 µM and 46 µM OPN compared to control treatment with PBS and to treatment with 460 µM caseinoglycomacropeptide (CGMP).
