# Supplementary figures and images for: Osteopontin adsorption to Gram-positive cells reduces adhesion forces and attachment to surfaces under flow
Source: J Oral Microbiol. 2017 Oct 11;9(1):1379826. doi: 10.1080/20002297.2017.1379826 (PMC5646589; doi:10.1080/20002297.2017.1379826)

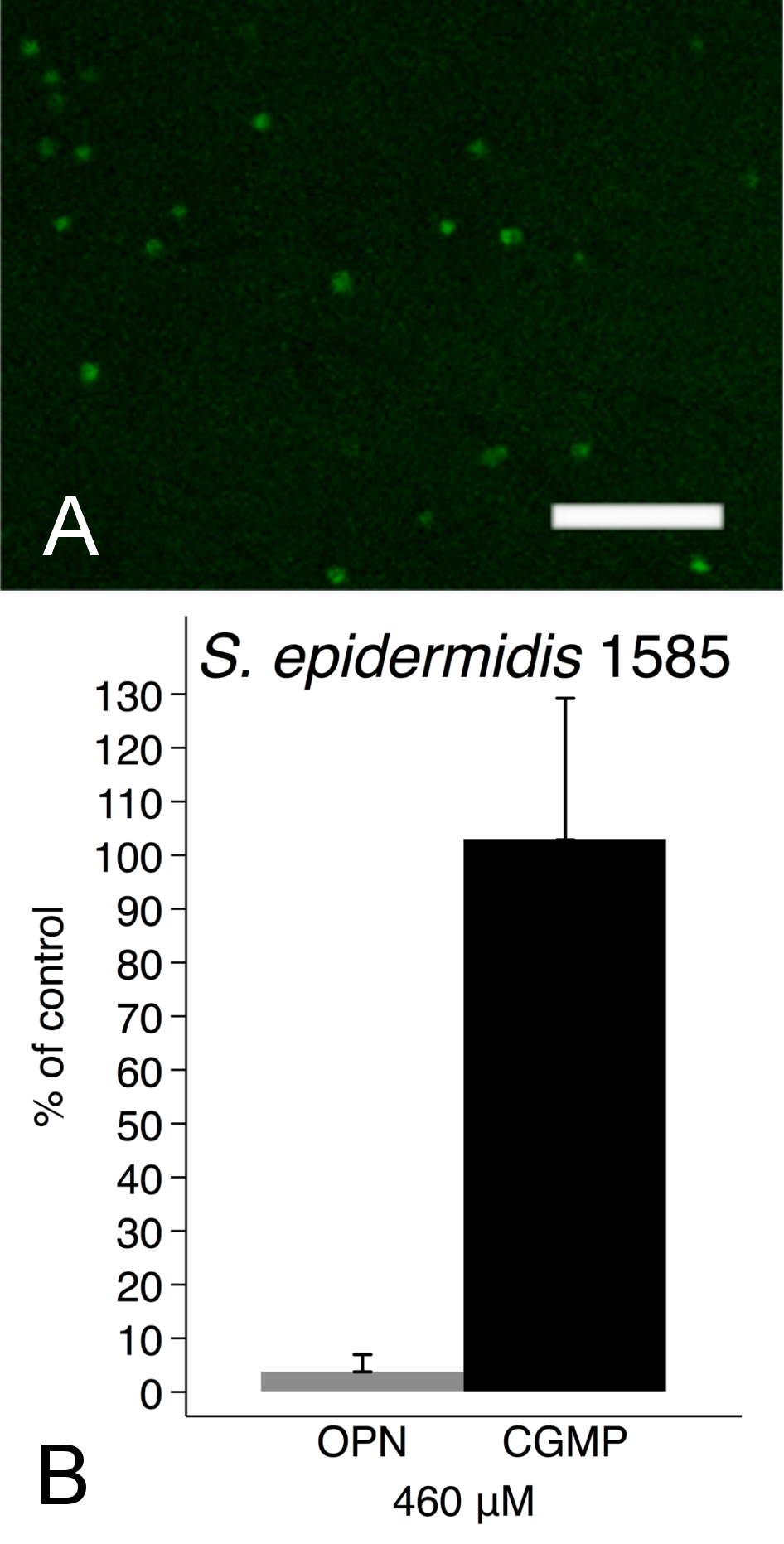

Supplement: Supplemenatal_data.zip [file ZJOM_A_1379826_SM6151.zip › Figure S2_NEW.tif]

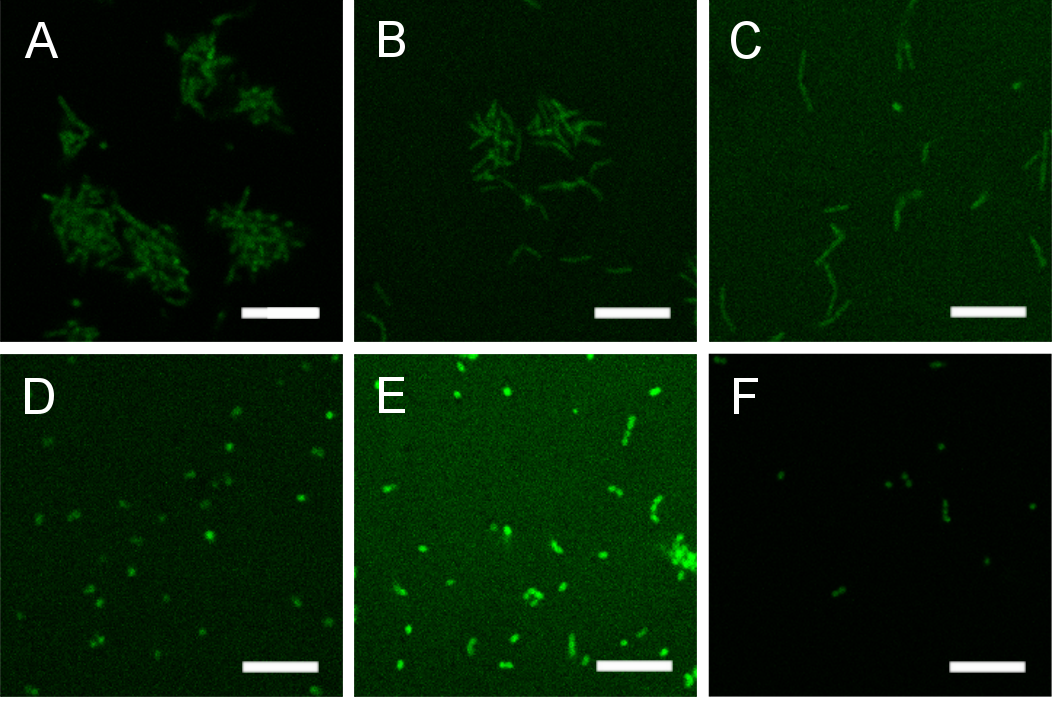

Supplement: Supplemenatal_data.zip [file ZJOM_A_1379826_SM6151.zip › Figure S1.tiff]
